# Electronic Noses and Tongues: Applications for the Food and Pharmaceutical Industries

**DOI:** 10.3390/s110504744

**Published:** 2011-05-02

**Authors:** Elizabeth A. Baldwin, Jinhe Bai, Anne Plotto, Sharon Dea

**Affiliations:** USDA-ARS Citrus & Subtropical Products Laboratory, 600 Ave S N.W., Winter Haven, FL 33881, USA; E-Mails: jinhe.bai@ars.usda.gov (J.B.); anne.plotto@ars.usda.gov (A.P.); sharon.dea@ars.usda.gov (S.D.)

**Keywords:** biosensors, chemical sensors, multivariate statistics, neural networks, pattern recognition, gas chromatography, mass spectroscopy, liquid chromatography, sensory, flavor, shelf life

## Abstract

The electronic nose (e-nose) is designed to crudely mimic the mammalian nose in that most contain sensors that non-selectively interact with odor molecules to produce some sort of signal that is then sent to a computer that uses multivariate statistics to determine patterns in the data. This pattern recognition is used to determine that one sample is similar or different from another based on headspace volatiles. There are different types of e-nose sensors including organic polymers, metal oxides, quartz crystal microbalance and even gas-chromatography (GC) or combined with mass spectroscopy (MS) can be used in a non-selective manner using chemical mass or patterns from a short GC column as an e-nose or “Z” nose. The electronic tongue reacts similarly to non-volatile compounds in a liquid. This review will concentrate on applications of e-nose and e-tongue technology for edible products and pharmaceutical uses.

## 1. Introduction

Electronic noses (e-noses) and electronic tongues (e-tongues) crudely mimic the human smell and taste sensors (gas and liquid sensors) and their communication with the human brain. The human olfactory system is by far the more complex and contains thousands of receptors that bind odor molecules and can detect some odors at parts per trillion levels [1] and include between 10 and 100 million receptors [2]. Apparently some of the receptors in the olfactory mucus can bind more than one odor molecule and in some cases one odor molecule can bind more than one receptor. This results in a mind-boggling amount of combinations that send unique signal patterns to the human brain. The brain then interprets these signals and makes a judgment and/or classification to identify the substance consumed, based in part, on previous experiences or neural network pattern recognition. The electronic nose often consists of non-selective sensors that interact with volatile molecules that result in a physical or chemical change that sends a signal to a computer which makes a classification based on a calibration and training process leading to pattern recognition. The non-selectivity of the sensors results in many possibilities for unique signal combinations, patterns or fingerprints. The human tongue contains sensors, in the form of 10,000 taste buds of 50–100 taste cells each [2], for sweet, sour, bitter, salty and umami and is much less complicated than the human olfactory system. The e-tongue then uses a range of sensors that respond to salts, acids, sugars, bitter compounds, *etc.* and sends signals to a computer for interpretation. The interpretation of the complex data sets from e-nose and e-tongue signals is accomplished by use of multivariate statistics including principal component analyses such as (PCA), linear discriminant analysis (LDA), discriminant function analysis (DFA), hierarchical cluster analysis (HCA), soft independent modeling of class analogy (SIMCA) and partial least squares (PLS). For non-linear responses, artificial neural networks (NAA) can be used for modeling the data. One must be careful, however, of false classification and over-fitting data, creating artificial differentiation especially in cases where the samples to variables ratio falls below six, when misclassifications can occur [3]. One approach to classify e-nose data, converted to principal components using (PCA), is to send as inputs to a support vector machine (SVM) or a relevance vector machine (RVM) classifier, which are new classes of learning algorithms [4].

Biosensors are also being developed, but are not yet commercialized. In contrast to chemical sensing materials, that are broad spectrum to generate characteristic response patterns, there are biological systems. The problem with chemical sensors is that these systems are extensive, require large sample sizes for analysis, have low sensitivity and poor specificity compared with the human nose. The bioelectronic nose utilizes olfactory receptors as sensing mechanisms and are cell or protein-based to mimic a mammalian olfactory system [5]. Another type of sensing system is based on colorimetric sensor array built in disposable chips [6]. These arrays are based on the chemical interactions between the analyte and a chemical dye. They are being developed for volatile [6–8] and non volatile molecules [8–10] for applications by the food industry.

The advantage of the human sensory system is that the brain can receive signals from both olfactory and tongue receptors and integrate both sets of data to form classifications and/or judgments. The e-nose and e-tongue are not integrated since each has its own software package, but the data from both instruments could be imported into another program and integrated. The disadvantage of the human sensory system is that no two brains are alike (of course from another point of view, this is a good thing), and the same brain may react differently from one day to the next, depending on an individual’s health, mood or environment, making the data subjective. On the contrary, e-nose and e-tongue instruments can be calibrated to be reliably consistent and can give objective data for important functions like quality and safety control. These instruments can also test samples that are unfit for human consumption. A disadvantage for the e-nose and e-tongue systems (as with humans) is that they are also affected by the environment including temperature for both e-nose and e-tongue and humidity for e-nose, which can cause sensor drift, although calibration systems and built-in algorithms help compensate for this. There are more or at least not less different types of sensing materials for e-tongue (liquid sensors) compared to e-nose systems, and liquid sensors often possess higher selectivity and significantly lower detection limits compared to the gas sensors (e-nose).

There are several reviews on the subject of e-nose and e-tongue technology, including reviews on e-noses [11,12], biomimetic/biotechnology e-nose and/or e-tongue sensing systems [5,13–15], applications for e-noses and e-tongues [2,16], neural networks for e-noses [17], pattern recognition techniques [18]; meat quality assessment by e-nose [19] and computational methods for analysis of e-nose data [20]. This review will concentrate on the recent literature on applications of e-noses and e-tongues in the food industry.

## 2. E-Noses

### 2.1. General Techniques

Metal oxide semiconductor (MOS), conducting polymer, and surface acoustic wave (transducers) are the most common e-nose sensors, [2,19]. The taste sensors for e-tongues are non-specific, low selective chemical sensors with cross-sensitivity to different components in solution [13]. However some e-tongue sensors are selective. Most e-nose and e-tongue instruments do not give information on sample composition, but rather give a digital fingerprint through pattern recognition. However, those instruments which use gas chromatography (GC) with solid phase microextraction (SPME) and/or mass detection by a mass spectrometer (MS) do give some information due to differences in chemical mass. A new e-nose on the market uses a short GC column which gives a crude chromatogram that can give information on odor molecules in the sample [21]. Calibration of e-noses and e-tongues is done with chemicals, but also by relating equipment responses to sensory data. It is only upon establishing a relationship to sensory perception that the e-nose and e-tongue instruments can then be substituted for sensory panels in giving objective classifications for quality control, process monitoring, authenticity, shelf-life stability and differences between samples or products.

Most e-nose instruments expose a limited number of sensors to volatiles, whereas a biological system uses a large number of sensors with diverse binding proteins. There are artificial systems that mimic biology such as the portable e-Mucosa System (PeM). This system utilizes three large sensor arrays, each with 200 chemoresistive sensors combined with two columns coated with different retentive layers (polar and non-polar compounds) giving pattern recognition that utilizes temporal information, improving the discrimination power of the instrument over traditional e-noses [22]. Another innovation is the Deferential e-nose (Den-nose) using two chemosensor arrays to discriminate odors [23]. The distance between the measured sensor signals, analyzed by two independent and identical sensor arrays is subjected to 2-dimentional convolution, which enhances sensitivity, allowing the ability to recognize similar odors. Sensor optimization is also important and is sometimes incorporated into e-nose data analysis software. Redundant signals due to cross-sensitivity of sensors can deteriorate classification performance and different methods to do this are reported [24]. For most e-nose systems, samples are analyzed using an array of gas sensors and a pattern recognition algorithm requiring exposure to odors and then flushing of the system for sensor recovery, and requires a chamber where the environment is controlled (temperature and humidity). Discrimination of odor in a real-world and dynamic environment results in sensors operating in a situation where they never reach steady state and the analysis is based on the transient phase of the signal [25] for real time identification, which is accomplished using tin dioxide gas sensors for continuous monitoring application. Zinc oxide, manganese oxide, titanium dioxide and tin dioxide are also reported to be useful when doped with silver ion in a sol-gel method or in naonstructured flat-type coplanar gas sensor arrays [26,27]. The MS can also be used as an e-nose and the headspace (HS) coupled with MS where the headspace of a sample is injected directly in the ionization chamber of the MS where they are fragmented resulting in a global mass spectrum for each sample giving a sample “fingerprint” as with other e-nose systems. Interference from ethanol, in the case of alcoholic beverages, can be mitigated by deleting the spectra fragments resulting from ethanol ionization [28].

### 2.2. Beverages

For wine, an e-nose with semiconductor oxide sensors were trained in tandem with a sensory panel [29] and used to asses enologists and provide early detection of some chemical compounds with the purpose of preventing wine defects. Alcoholic beverages are difficult to analyze by e-nose due to the ethanol and carbon dioxide (beer and sparkling wines). A French manufacturer e-nose, AlphaMOS e-nose (FOX 4000), along with PCA and DFA analyses were used to discriminate beer and wines tainted with off-flavors (1-hexanol, ethyl acetate, 4-ethylphenol, octenol, and 2,3,4-trichloroanisole (TCA) after dehydration and dealcoholization of the samples [30]. The same e-nose was useful for characterizing different fruit and grape wines produced in Ontario, Canada [31] based on their odor profiles including blackberry, cherry, raspberry, blackcurrant, elderberry, cranberry, apple and peach, as well as red, Chardonnay, Riesling and ice (grape) wines. Separation was possible based on winery, but when wines were combined, classification based on variety was poor. Fruit and grape wines were well separated as were red and white grape wines. The authors propose that the e-nose may have ability to elucidate the relationship between wines, and may be useful for quality and uniformity control. An HS-MS system was used for calibration transfer applied to the analysis of wine aroma using synthetic wine prepared from the most common wine aromas (ethyl hexanoate, isoamyl acetate and 2-methyl butanol). The signal variations of the synthetic wines over time was considered representative of those in real wines, enabling an accurate correction [28]. Geographical origin of Sauvignon Blanc wines, from which the ethanol was removed, from three countries and six regions was resolved by GC-MS that was then used to train a MOS e-nose and a HS-MS e-nose with LDA [32]. A similar study was done using HS-MS with Tempranaillo wines which were classified according to geographic origin using chemometrics (PCA, PLA and stepwise linear discriminant analysis, SLDA). The data from the HS-MS was exported into the Unscrambler software for the chemometric analysis [33]. The SLDA correctly classified 86% of the samples, while the PLS correctly classified 85%. In another study, wine samples were enriched using purge and trap and SPME then applied to a custom designed e-nose with a tin oxide array for discrimination [34]. Making must is the first step in winemaking and has high sugar content. Musts from off-vine dried grapes were analyzed by a quartz microbalance (QMB) e-nose developed at the University of Rome Tor Vergata. The e-nose could distinguish between fermented and unfermented musts, but not between wines fermented by different *Saccharomyces cerevisiae* strains [35].

For tea, usually analyzed using a GC and sensory panel, an e-nose (a Shimadzu FF-2A Fragrance & Flavor Analyzer) was used to identify coumarin-enriched Japanese green tea using PCA and cluster analysis (CA) [36]. The study also used the e-nose to determine the appropriate temperature and infusion time for emission of coumarin-like flavor. Using a newly developed “absolute value expression” (AVE) method, the authors were able to divide tea flavors into quality categories, expressed numerically, and the role of coumarin in tea flavor was determined.

Coffee quality is in practice evaluated by expert tasters (“cup tests”), or by GC-MS looking for aliphatic hydrocarbons derived from oxidation of green bean lipids during storage or transport prior to roasting. Cup tests are subjective and not always reproducible, depending on the skills and training of tasters. Therefore, e-nose methods would help industry in QA-QC. An array of 12 tin oxide sensors was able to classify 90 samples of coffee consisting of two blends and two roasts with an 81.1 to 95.5% success rate and also could tell differences due to roasting time [37]. An e-nose (University of Pamplona, Colombia “A-NOSE” with metal oxide sensors) was also used to classify Colombian coffee for defects in “cup” tests [38] for quality control using PCA and validation using ANN. Colorimetric disposable sensor arrays were able to consistently discriminate among commercial coffees (Suslick *et al.*, 2010). The same arrays were able to discriminate roasting time (1 min to 3 h at 220 °C) and roasting temperatures (180 to 240 °C). In another study, headspace from espresso coffee samples were analyzed by a low fragmentation, high time resolution, broad detection range MS (proton transfer reaction MS or PTR-MS gas analyzer with chemical ionization) with a “hot liquid headspace inlet system” [39] coupled with a GC/MS with electron ionization for ambiguous identification. This data was correlated to trained sensory panel data to build a model based on sensory and PTR-MS data to predict the sensory profile of the coffee samples based on fast on-line PTR-MS analysis opening the possibility for high through-put studies.

**Figure 1.**
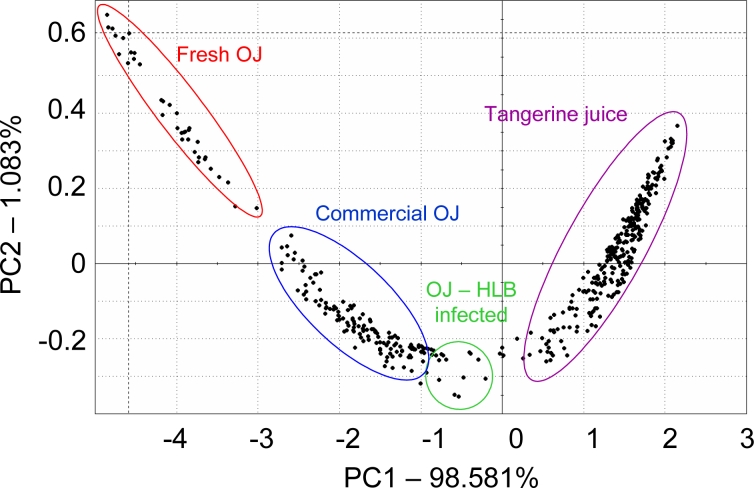
PCA plot of citrus juices based on the electronic nose signals. The observations are grouped by juice type, fresh squeezed orange juice (OJ) with high peel oil, processed OJ, processed OJ from Huanglongbing (HLB) infected fruit, and fresh squeezed tangerine juice.

For citrus juice, a French Alpha MOS e-nose and headspace volatile analysis using SPME were used to group 76 commercial and 120 self-prepared citrus juices according to fruit type, cultivar and treatment using LDA. In one case, a commercial orange juice grouped close to grapefruit and two declared grapefruit juices ended up being miss-assigned to grapefruit and were in fact pummelo, exposing wrong or misleading supplier information and human error [40]. In another study with the French Alpha MOS e-nose, not-from-concentrate (NFC) orange juice was separated from frozen concentrated orange juice [41]. A study from our laboratory looked at the Alpha MOS e-nose with 18 sensors, for ability to separate orange juice from fresh squeezed oranges, orange juice from a simulated commercial process (including pasteurization), orange juice from fruit harvested from healthy trees and the same commercially processed juice made from fruit harvested from Huanglongbing (HLB) infected trees and fresh squeezed tangerine juice (Figure 1). The e-nose separated all these juices using PCA, even the juice from HLB-infected trees, which were shown to have fruit that have off-aroma and a bitter-metallic flavor [42,43].

### 2.3. Grains

An e-nose technique was optimized to classify wheat based on storage age [44,45] using PCA and LDA and an Airsense analytics PEN2 nose with metal oxide semiconductors. Fungal volatiles of naturally infected and inoculated (*Fusarium culmorum*) wheat and triticale grain were analyzed by e-nose (Perkin Elmer TurboMatrix HX40 headspace analyzer and TurboMass MS as a detector). All samples contained varied levels of trichodiene, a precursor to fusarium metabolites, with six fold higher levels in inoculated samples. Triticale grain could also be separated from wheat [46]. The e-nose was also used to detect key aromas that related to the different stages of the bread-baking process [47] using a 4-sensor array (thin films based on titanium dioxide, mixed molybdenum and tungsten oxides and indium oxide). The Cyranose 320 with 32 polymer sensors was able to distinguish between varieties of long grain rice [48].

### 2.4. Cooking Oils

An e-nose with six metal oxide sensors (Italian EOS 507, Sacmi Imola, SC), was used to classify virgin olive oils with and without phenolic compounds for oxidative status [49] and correlated well to sensory analysis. Two types of e-nose (Alpha MOS and SPME-MS), along with PCA and PLS analyses, were also able to detect adulteration of extra virgin olive oil with rapeseed and sunflower oils [50]. Likewise adulteration of virgin coconut oil with palm kernel olein (adulterant volatile methyl dodecanoate) was detectable using a Z-nose based on surface acoustic wave sensor technology [51].

### 2.5. Eggs and Dairy Products

An e-nose could distinguish eggs stored for different amounts of time and at chilled or room temperature storage [52] using PCA and LDA analyses combined with neural network. An ion-mobility based e-nose (MGD-1) was used to determine separation of hard and extra-hard cheese samples as well as discrimination of cheeses based on age (ripening time) or origin [53]. Ion mobility spectrometry (IMS) allows rapid on-site determination of volatiles by hand-held devices by ionization of gas molecules. An Alpha Mos E-nose with metal oxide sensors was also used to determine shelf life of milk [54] at ambient or refrigerated temperatures as well as bacteria growth in the milk. The French Alpha MOS e-nose was used to determine differences in milk flavorings including three natural flavorings and two synthetic as well as one self-made enzyme-induced milk flavoring prepared by lyophilized milk fat [55]. Using PCA, the e-nose could distinguish the difference among all the milk flavorings as well as between the natural and enzyme-induced milk flavorings that were not that distinguishable in sensory tests. These discriminations were confirmed by SPME GC-MS.

### 2.6. Meat and Fish

For meat, the e-nose has been used to detect bacterial spoilage during the aging process using biosensors that included a silver or platinum electrode on which the enzymes putrescine or xanthine oxidases were immobilized [19]. For meats, sensory quality, shelf life spoilage, off-flavor, taints and authenticity are areas where volatile changes make e-nose screening of samples useful [19]. A KAMINA e-nose with a MOS microarray and LDA was used to evaluate pork meat freshness when stored at 4 and 25 °C. Three e-noses were operated in parallel for statistical reliability of results. Three to four training cycles were required to build a reliable LDA model. An e-nose (Pen-2 model with MOS sensors) was used to distinguish between fresh and “old” minced beef samples, determining the range of stability time at specific temperatures in modified (high oxygen) atmosphere packaging (MAP) [56]. An e-nose system with metal oxide sensors was used to detect changes in the headspace of stored beef strip loins inoculated with Salmonella typhimurium. The principal components and independent components were plotted against Salmonella population counts and a stepwise linear regression model built [57]. An e-nose could distinguish broiler chicken in MAP packages with deteriorating quality from fresh in conjunction with sensory quality changes and was consistent with certain microbial counts [58]. A MOS based e-nose was used to discriminate between human pathogens *E. coli* and *Listeria* [59].

For fish, freshness was determined by measuring the relevant volatile compounds consisting of alcohols, carbonyls, amines and mercaptanes which showed typical concentration changes over time under specific storage conditions [60] using amperometric sensors, a heated catalyst and multivariate statistics (PCA and principal components regression or PCR). E-nose (PEN-2 model) with MOS sensors was used to predict the freshness shelf life of sea bass using a time/temperature storage regime [61]. Another e-nose with a micro-machined metal oxide array was used to assess sardine levels of freshness and was compared to SPME analysis of headspace for identification and quantification of compounds and to predict total viable counts of aerobic bacteria present in the samples [62]. Formaldehyde is sometimes illegally used to prevent spoilage of seafood, which is a danger to consumer health. An e-nose with TGS (Japanese Taguchi gas sensor) was used to distinguish between water and formaldehyde-dipped octopus [63] as a determination of spoilage.

### 2.7. Fresh, Fresh-Cut and Processed Fruits and Vegetables

For stone fruit, an Alpha-MOS E-nose was able to discriminate between different varieties of apricot using PCA and factorial discriminate analysis (FDA) and then was compared to a classification of the same varieties by measurement of aroma compounds by SPME GC-MS [64]. An Italian EOS835 e-nose, with six metal oxide sensors, was also used to follow aroma development during ripening and storage of apricots at harvest, after 15–30 days storage at 0 °C and after simulated shelf life at 20 °C. As with the other study, the e-nose classification was compared to measurement of aroma volatiles by GC-MS and sensory analyses [65]. A portable PEN2 German e-nose with metal oxide sensors was used to classify four peach cultivars and to assess and monitor ripening stages using PCA, LDA and “classification and regression tree” (CART). In this case, one to three sensors out of ten explained most of the variation. This was compared to other ripening detection techniques such as measurement of ethylene or color, and some correlations were found.

For mangoes, classification and differentiation has been achieved with e-nose technology. A French Alpha MOS e-nose was used to classify mango homogenate as well as whole fruit headspace for variety differences, harvest maturity, fruit size and ripening stage [66]. This was compared to using a GC for classification. Generally, the e-nose was more successful in classifying the mango samples. The zNose™, which has a short, fast GC column and an uncoated surface acoustic wave sensor (transducer), was used to determine occurrence of decay and ripeness of the mango fruit. Two peaks detected by the zNose™ correlated to decay and another showed accuracy in predicting ripeness in relation to color [67]. Peaks important for prediction of decay or ripening were determined by PLS combined with “variable importance for projection” (VIP).

For apples, a Cyranose with 32 composite polymer sensors was used in conjunction with a zNose™ to improve classification of damaged apples. The combination of technologies was compared to use of either technology alone [68]. This was accomplished using feature level and decision level multi-sensor data fusion models and covariance matrix adaptation evolutionary strategy (CMAES) that was developed to fuse the e-nose and zNose data [68]. Fresh-cut apple slices pretreated with heat and ethanol had reduced/altered flavor by sensory panel [69]. Canonical discriminant anlysis of headspace GC and Alpha MOS e-nose equally separated the treated samples from the control.

For blueberries, a Cyranose was used to detect and classify diseased blueberry fruit inoculated with grey mold (*Botrytis cinerea*), anthracnose (*Colletotrichum gloeosporioides*) and *Alternaria* rot (*Alternaria* sp.) [70]. Volatiles resulting from inoculations were resolved into four groups (including non-inoculated control) using PCA.

For grapes, GC-MS and an e-nose was used to monitor postharvest management of water loss since this can contribute to modification of the volatile profile and affect subsequent wine quality. The e-nose used was developed at the University of Rome Tor Vergata and was based on an array of eight quartz microbalances (QMB) which were exposed to headspace samples [71]. The e-nose could determine differences (confirmed by GC-MS) due to temperature and time following the dehydration process.

For dates, an Alpha Mos e-nose was used to distinguish between varieties using PCA, and gave distinct fingerprints for each variety that could be used following changes in maturity and identify lots of dates as well as adulteration [66]. The e-nose showed excellent sensitivity and reproducibility, which was necessary since the date volatile profile is weak.

For pineapple, fresh cut pineapple was followed using a portable German PEN2 e-nose with MOS sensors during storage at three different temperatures using a continuous (automatic sampling of headspace air flow) and discontinuous (headspace samples taken at various times throughout storage) approach [72]. The discontinuous approach was able to discriminate between samples due to volatiles associated with quality and decay using PCA and CA. From this data the authors were able to determine stability time and derived a time/temperature tolerance chart by modeling the aroma change of the fresh-cut pineapple as a function of storage time and temperature.

For tomato, different e-nose instruments including the German PEN2 and one with organic polymer sensors were able to distinguish different ripening stages between green stages [73] and ripe [74] using PCA LDA and DFA. One study tried to determine ripeness in a carton box and folded bag [74], and the e-nose was better able to discriminate ripening in the box than in the bag. GC-MS of headspace of spoiled tomatoes showed changes in volatile compounds due to bacteria, yeast and fungi within a few hours of contamination [75]. An e-nose (Italian EOS835 with metal oxide sensors) was then shown to be able to reveal contamination at early stages of canned, peeled tomato using exploratory data analysis (EDA) software that includes PCA.

## 3. E-Tongue

E-tongues have reportedly been used to obtain data for sourness, bitterness and astringency for foodstuffs such as beers, wines and teas [76–79]. This involved detecting polyphenols and predicting sensory attributes of bitter, sweet, sour, fruity, caramel, artificial, burnt, intensity and body using potentiometric/amperometric chemical sensors along with the same pattern recognition techniques described above for e-nose technology. Taste sensation is the result of physico-chemical interactions of food molecules with a complex system of hundreds of cell buds located randomly all over the tongue [16]. The principle for the e-tongue is to combine signals from specific, non-specific and overlapping sensors with pattern recognition as has been described for the e-nose. For amperometric sensors there are four classes including metal, conducting polymer, phtalocyanine film and biosensors. Nanocomposite materials are still being developed. Three different detection modes are described including fixed potential preferred in flow systems and for biosensors, step pulse potential and sweeping potential of which the last two are preferred in batch systems. Up and coming are microchip capillary electrophoresis coupled with amperometric screen printed electrodes [16]. Metal sensors are also used, but lack selectivity. They are more useful for classification applications than for evaluation of taste, like predicting sensorial descriptors of Italian red dry wines of different origins [80]. Conducting polymer sensors such as polypyrrole and polyaniline show a variation of conductivity with adsorption of different analytes [16]. E-tongues based on conducting polymers have been used to evaluate bitterness [81], as well as sweet, bitter, acid, salty and astringent tastes [82]. The advantage of conducting polymers is the rapid adsorption/desorption and partial selectivity through modifying the dopant of the polymer, however they are sensitive to humidity (problem for e-nose but not e-tongue that analyzes liquids) [16]. Phtalocyanine film electrodes can have different chemical properties. These are coordination compounds where a transition metal is coordinated with a phthalocyanine ring. Films of phthalocyanine, porphyrin and naphthalocyanine showed cross-selectivity to antioxidant compounds like banillic acid, progallol, ascorbic acid and catechin [83]. E-tongues based on films of phthalocyanine sensors could discriminate between model solutions of sweet, bitter, salty, acid an umami basic tastes [84] and bitterness in olive oils [81]. Biosensors for e-tongue are systems with a biochemical transducer, an enzyme and solid electrode in intimate proximity. Enzymes are oxidases that consume oxygen and produce hydrogen peroxide or the reduced form of β-nicotinamide adenine dinucleotide (phosphate) NAD(P)H as a dehydrogenase [16]. Improvement in performance of metal sensors, conductive polymers or biosensors is linked to scaling down of size to nanodimensions which increases the surface to volume ratio of the sensors, lowering detection limits [16].

New techniques are being applied to develop miniaturized sensor arrays such as screen printing for thick-film and electron beam evaporation, thermal vacuum deposition and pulsed laser deposition for thin-film technique [85]. Micro-fabrication techniques were used to prepare a sensor array for use in a voltammetric e-tongue by depositing gold (Au), platinum (Pt), iridium (Ir) and rhodium (Rh) on a silicon wafer [85]. Colorimetric sensor array for non-volatile molecules are still under development. These are made from nanoporous pigments immobilized onto porous modified silicate and printed on a hydrophilic membrane [9].

E-tongue sensors often use a lipid membrane as a recognition element that changes taste relevant substances into electric potential charge across thin membranes and the membrane potential is independent of thickness of the lipid membrane, which must have durability and reproducibility even when repeatedly rinsed [86]. The concentration of the lipid in the membrane can be optimized and affects the detection limit of the sensor to adsorptive taste substances [86].

### 3.1. Beverages

For wine, a custom-designed e-tongue with a hybrid sensor array consisting of voltammetric electrodes modified chemically with different electro-active substances (polymerized aqueous solution of pyrrole using six doping agents) was used to discriminate and recognize among 12 Spanish red wines based on denomination, origin, grape variety and vintage [87] due to the cross-selectivity of the electrodes. This was accomplished using PCA, PLS discriminant analysis (DA) and SIMCA analysis. Compatibility of wine with fish *versus* sake was explored using a Japanese taste sensor system along with sensory [88] using a SA402B taste sensor system fitted with five sensor probes and two reference probes (lipid-polymer membrane probes with silver/silver chloride (Ag/Ag/Cl) electrodes and an internal cavity filled with a 3.3 M potassium chloride (KCl) solution saturated with AgCl). An e-tongue comprised of a multisensor system of 26 potentiometric chemical sensors with sensitivity to organic anions, phenols, organic cations and a pH electrode *versus* a conventional Ag/AgCl reference electrode was used as a rapid analytical tool for wine age prediction in comparison to high pressure liquid chromatography (HPLC) with respect to organic acids, phenolics and furanic derivatives in combination with ANOVA-Simultaneous Component Analysis (ASCA) [79]. The sensor system was developed at St. Petersburg University and was capable of predicting concentration of tartaric, citric, formic, protocatehuic, vanillic and sinapic acids, catechin, vanillin and trans-resveratrol.

For beer, the same type sensor system as used for predicting wine age was used to characterize different types of beers including lager beers, ales and wheat beers among others and relating the data to sensory studies. The e-tongue was capable of predicting 20 sensory attributes of beer including bitter, sweet, sour, fruity, caramel, artificial and burnt [79]. In another study of fifty Belgian and Dutch beers were studied for physiochemical parameters such as real extract, real fermentation degree, alcohol content, pH, bitterness, color, polyphenol and carbon dioxide content using Canonical Correlation Analysis (CCA) [77]. Bitterness in beer is an important quality parameter coming mainly from the hops forming bitter iso-α-acids. This e-tongue system proved capable of predicting real extract, alcohol and polyphenol content as well as bitterness.

For tea, an e-tongue along with multivariate calibration (PCA-ANN) was able to determine contents of catechins and caffeine in green tea [89] in comparison to reverse phase HPLC. The taste system consisted of seven silicon transistor sensors with an organic coating and an Ag/AgCl reference electrode. The response intensity of each sensor was measured by the voltage difference between each coated sensor and the reference electrode. Another taste sensor system of non-specific solid state potentiometric sensors along with PCA was used to differentiate between tea samples from different geographic regions and quality grades [90]. This information was compared to ten sensory attributes of tea taste and it was determined that the e-tongue could predict sensory characteristics and their relationship to tea flavor quality.

For milk, a potentiometric e-tongue and PLS-DA analysis was able to classify milk based on producer and origin using an array of microelectrodes (Ag/AgCl) fabricated from epoxy-glass laminate and polyvinyl chloride (PVC) membranes with various additives as the chemosensitive layers [91]. In another study a potentiometric sensor array was used to monitor changes in probiotic fermented milk during storage and to accurately predict results form a sensory panel. The e-tongue system consisted of seven sensors and an Ag/AgCl reference electrode [92]. PCA was used to monitor changes occurring in the milk, ANN for classification of milk during storage and PLS and ANN to estimate and predict sensory characteristics. Adulteration of milk (with hydrogen peroxide) was detected by a disposable voltammetric e-tongue (Dutch PalmSens potentiostat) with gold and copper sheet substrates and sensing elements of gold, copper and gold surface modified with a layer of Prussian Blue [93]. An e-tongue with 36 cross-sensibility sensors showed high sensibility to acid, salty and umami tastes, but had lower recognition of bitter and sweet tastes. The system was, however, able to detect goat milk adulteration with bovine milk [94]. Different profiles recorded for raw skim milk from goat, cow or goat/cow was demonstrated using LDA.

For fruit-based soft drinks, a similar potentiometric e-tongue with 36 cross-sensibility lipid/polymeric membranes was used to test commercial fruit juices (orange, pineapple, mango and peach) from different brands [95]. The authors used LDA to differentiate four beverage groups based on fruit juice content. The signals were also used to obtain multiple linear regression and PLS calibration models to estimate/predict sugar concentrations. Another study with a similar system was able to differentiate non-alcoholic beverage groups with different added fruit juice contents using stepwise LDA [96].

**Figure 2.**
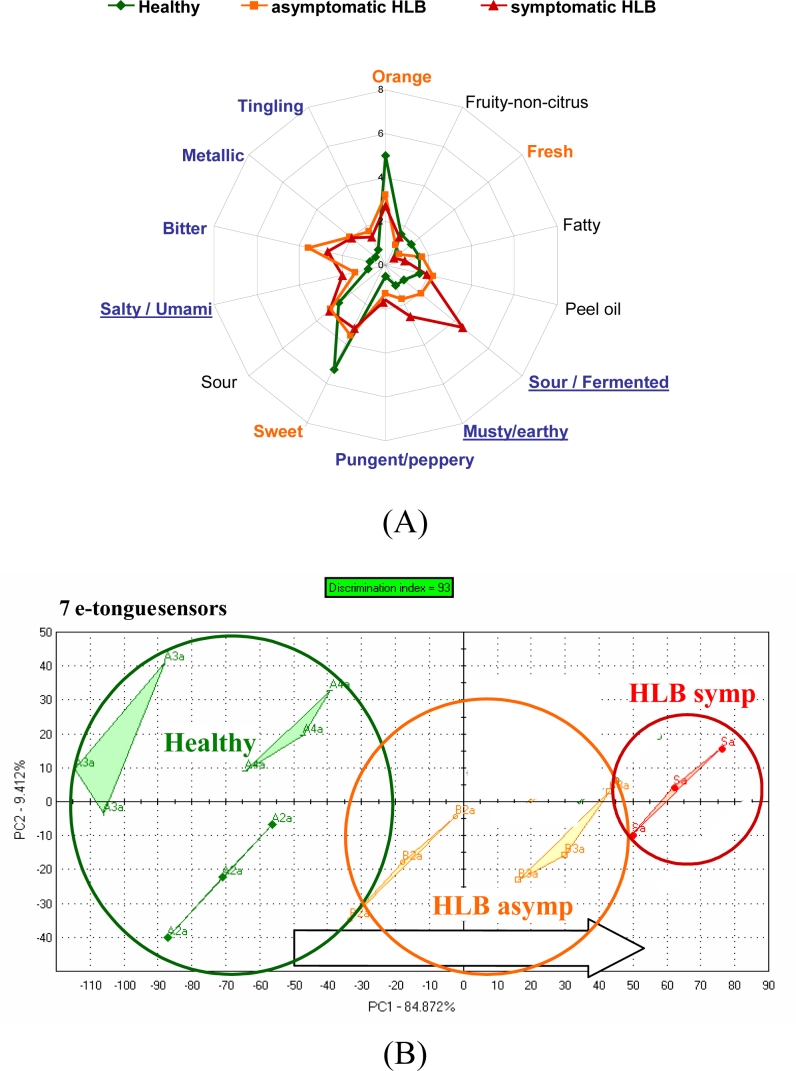
**(A)** Trained sensory panel rating of processed juice from Hamlin oranges harvested from healthy or Huanglongbing (HLB) diseased trees (2008) including juice from asymptomatic (normal looking) and symptomatic fruit (symptomatic for the disease: small, green and lopsided). Healthy juice was significantly higher in orange flavor, fresh and sweet tastes, and HLB juice was higher in sour/fermented, musty/earthy and salty/umami tastes. **(B)** E-tongue (AlphaMOS ASTREE) PCA plot of the same juice.

For orange juice, an integrated array of solid-state ion-sensitive microelectrodes as used to construct an e-tongue. The ion-sensitive sensor array was obtained by deposition of PVC membranes with different sensitivities towards various ionic species, forming a miniaturized e-tongue system which was capable of recognizing brands of orange juice [97] using PLS as a classifier. Juice from fresh sweet oranges with differing limonin content (bitter compound) due to the delayed bitterness phemomenon, were positively correlated with the relative bitterness value measured by an Astree (Alpha MOS) e-tongue equipped with a #5 sensor set [98]. In a study from our lab, the Alpha MOS Astree e-tongue was able to separate between juices of processed orange juice from fruit harvested from healthy trees and those harvested from HLB-infected trees which were symptomatic for the disease (small, green and lopsided) or asymptomatic (normal looking fruit). A trained sensory panel showed differences between juice from healthy trees and juice from fruit harvested from HLB-infected trees [Figure 2(a)].

**Figure 3.**
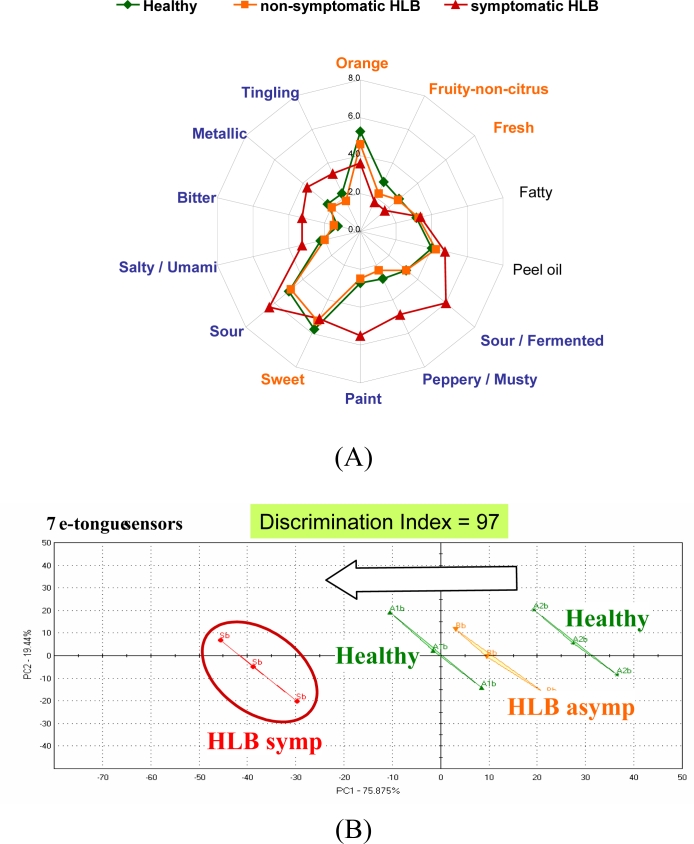
**(A)** Trained sensory panel rating of processed juice from Hamlin oranges harvested from healthy or Huanglongbing (HLB) diseased trees (2009) including juice from asymptomatic (normal looking) and symptomatic fruit (symptomatic for the disease: small, green and lopsided). HLB symptomatic juice was significantly lower in orange flavor, fruity non-citrus, fresh and sweet tastes and higher in sourness and off flavors than asymptomatic or healthy juices, which were not different from each other. **(B)** E-tongue (AlphaMOS ASTREE) PCA plot of the same juice.

**Figure 4.**
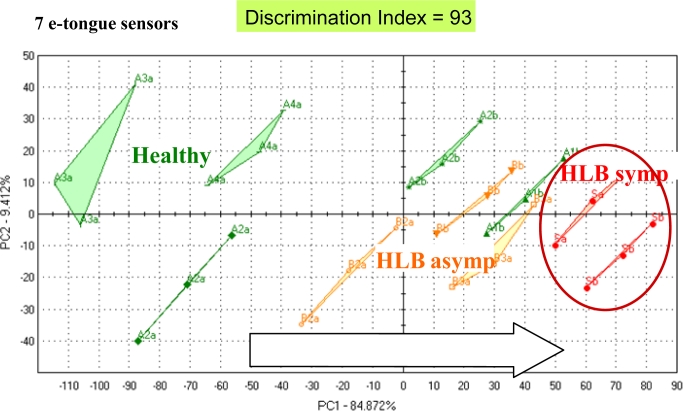
E-tongue (AlphaMOS ASTREE) PCA plot of Hamlin orange juice processed in 2008 and 2009, from fruit harvested from healthy or from Huanglongbing (HLB) diseased trees including juice from asymptomatic (normal looking) and symptomatic fruit (symptomatic for the disease: small, green and lopsided).

Differences were highest for orange flavor and sweetness. Furthermore, sour/fermented, musty/earthy and salty/umami descriptors were rated higher for juice from fruit symptomatic for the disease [Figure 2(a)]. The juice from these fruit was later found to be higher in the bitter compounds limonin and nomilin and lower in sugars [42] compared to healthy or asymptomatic HLB juice. The e-tongue separated the samples similarly to the sensory panel with all three groups segregating [Figure 2(b)]. In another season, the same panel could not distinguish between juice from healthy fruit and juice from asymptomatic HLB fruit, but did rate sourness and many off flavors significantly higher for juice from HLB symptomatic fruit [Figure 3(a)]. The e-tongue separations were similar to the panel in that only juice from HLB-symptomatic fruit were segregated from healthy, while juice from healthy fruit could not be separated from HLB asymptomatic fruit juice [Figure 3(b)]. When combining both years, juice made with symptomatic fruit from HLB infected trees were again well separated from juice from healthy or normal looking fruit from diseased trees (Figure 4).

For tonic water, a piezoelectric quartz crystal (PQC) sensor array based on molecularly imprinted polymer (MIP) coating was developed for sensing quinine and saccharine in bitter drinks like tonic water. The MIP-PQC sensor array could detect changes in bitterness and the negligible suppressing effect of saccharine in comparison to a sensory panel [99].

### 3.2. Fruit and Vegetables

For tomato, two e-tongue systems were evaluated for ability to analyze taste; an e-tongue developed at the University of St. Petersburg with 18 potentiometric sensors and the Alpha MOS Astree e-tongue with seven sensors [100]. Tomato cultivars were selected based on trained sensory panel data on differences in sweetness and sourness. Chemical measurements of sugars (glucose and fructose), acids (citric, malic and glutamic) and certain minerals (Na and K) were made. Data were analyzed using PCA canonical discriminant analysis (CDA) and PLS to classify the cultivars based on taste profile and to quantitatively relate the taste components to sensory panel scores. Both e-tongue systems did well, but the St. Petersburg system could not predict general sweetness and umami taste compared to panel data, while the Alpha MOS Astree was better at quantifying glutamic acid and Na, but not so good at classifying sweetness sourness, saltiness and umami.

### 3.3. Cooking Oils

For oils, a microelectrode was used as an e-tongue along with application of chemometrics and was useful for discriminating oils based on their quality and geographic origin [101]. It was necessary to add suitable room temperature ionic liquids to the oils as supporting electrolytes to provide conductivity to the otherwise low-polarity samples. Using PCA and a classification technique (K nearest neighbors or KNN), the system was able to differentiate different oils (maize and olive) or geographical origin (olive oils from different regions).

For honey, a French α-Astree (Alpha MOS) e-tongue with seven potentiometric chemical sensors was used to classify honey samples from different floral and geographic origins [102] using PCA, CA, and ANN. Each sensor was composed of an organic coating sensitive to the spice in the samples and a transducer to convert the response of the membrane into a signal (change in voltage intensity between the chemical sensor and the Ag/AgCl reference electrode).

### 3.4. Meat and Fish

For meat, a method was developed for predicting levels of sodium chloride (NaCl), sodium nitrate (NaNO2) and potassium nitrate (KNO3) in minced meat using two different electrochemical methods: an e-tongue based on pulse voltammetry and electrochemical impedance spectroscopy [103]. Both methodologies did well in NaCl determinations while prediction of nitrite and nitrate was not as good. Sensor optimization and combining the data collected from both electrochemical methods improved predictive capabilities for NaCl and NaNO_2_.

For fish, an e-tongue of simple potentiometric electrodes (Au and Ag wires measuring variation of Au an Ag potentials) could determine the post-mortem time elapsed in minced gilthead sea bream using simple potentiometric measurements relating to fish freshness [104]. Validation was achieved by analysis of concentration of certain bio-molecules as a function of time after death such as ATP metabolites inosine 5′-phosphate (IMP), inosine (Ino) and hypoxanthine (Hx).

### 3.5. Pharmaceuticals

For pharmaceutical products, the same Japanese e-tongue with seven lipid membrane sensors was used to detect bitterness of quinine hydrochloride, one of the more bitter drugs. The study found a low detection limit for quinine hydrochloride comparable to human taste sensation [105]. A French Alpha MOS Astree II e-tongue with seven sensors (the pharmaceutical sensor set with chemically modified field effect transistors), was used to evaluate bitterness in a solution of a drug in water and several marketed products to determine taste-masking effectiveness of formulations compared to placebos. This was correlated to sensory data for the purpose of evaluating unknown formulations to predict their bitterness [106] using a PLS regression curve, and formulation masking effects. Tested formulations consisted of a drug in an aqueous-based solution with sorbitol, citric acid, sodium citrate, artificial cherry flavor (unless unflavored prototype) and sodium benzoate. Some samples contained an artificial sweetener compared to high fructose corn syrup, and it was found that the artificial sweetener better masked the taste of the drug.

## 4. Mixed Technologies (E-Tongue, E-Nose and E-Eye)

Prediction of sensory characteristics and their relationship to quality of apple juices was accomplished using an Alpha MOS α-ASTREE e-tongue and a Prometheus e-nose and quantitative descriptive analysis (QDA). The authors determined that these instruments together could be used to track consumer-defined quality if the appropriate compounds and measures were defined, using a trained sensory panel [107]. An electronic panel (e-nose, e-tongue and e-eye) was used to characterize organoleptic characteristics of virgin olive oil samples from different olive varieties for degree of bitterness [108]. It was found that the discrimination of the combined system (using PCA) was better than any of the three systems alone. PLS regression models showed good correlations between the e-tongue signals and the polyphenolic content and the bitterness index produced by sensory panelists.

## 5. Importance of Sensory Information for Training of E-Nose and E-Tongue

The potential use of e-nose and e-tongue technology is desirable for fast analysis of samples to replace or complement sensory panels. Sensory panels may not always be available, or QC personnel may not be consistent in evaluating samples, hence the urge to use fast and reliable instrumental techniques. In an ideal world, specifications are determined by trained and consumer panels using preference mapping [109], and the electronic sensors are calibrated to a set of criteria that correlate with sensory data [107]. Many studies reviewed herein have shown correlations between electronic sensors and taste panels. Some studies revealed that there is still some disparity between instruments, most of which are able to discriminate and classify samples, but classifications do not always correlate with sensory data [65,100]. Nevertheless, the approach of using the combination of e-nose and e-tongue, and even e-eye seemed to result in better correlations with sensory data [105,106]. Like the processing of complex sensory information (stimuli to human sensory system), this multi-sensors approach would more closely reflect the complexity of human perception.

In the goal of masking bitterness in product development [99,106], it was possible to calibrate e-tongues and overcome the use of a taste panel. In this situation however, one needs to remember that bitterness perception is highly variable among individuals, and that the variation can differ for one bitter compound compared to another or compared to multiple bitter compounds [110]. The calibration of the sensors must be done under careful conditions.

Also e-nose is useful when comparing treatments not approved for human consumption. For example, a MOS e-nose was able to separate fresh-cut apples treated with different compounds to reduce browning, one of which had not yet been approved for human consumption. Sensory panels were used to differentiate between treatments that were approved for human consumption. This data was also correlated to flavor chemicals such as aromas [69]. Between the e-nose and the sensory data, a comprehensive picture of treatment effects was achieved.

## 6. Conclusions

The development of artificial senses technologies is occurring rapidly, with demonstrated ability to differentiate among food and edible products for aroma, bitterness, and other basic tastes. The systems are becoming faster, more reproducible and smaller. What is needed is speed, reproducibility, consistency and robustness for commercial applications. Likewise data analysis systems are being developed and applied to these artificial sensing systems, to integrate responses with sensory and chemical data and to combine data of different technologies (like e-noses and e-tongues) to better replicate the human sensing system.
